# Monitoring the prevalence of thyroid disorders in the adult population of Northeast Germany

**DOI:** 10.1186/s12963-016-0111-3

**Published:** 2016-11-08

**Authors:** Rehman Mehmood Khattak, Till Ittermann, Matthias Nauck, Harald Below, Henry Völzke

**Affiliations:** 1 Institute for Community Medicine, Ernst Moritz Arndt University, Walther Rathenau Str. 48, 17475 Greifswald, Germany; 2 Institute of Clinical Chemistry and Laboratory Medicine, Ernst Moritz Arndt University, Greifswald, Germany; 3 Institute of Hygiene and Environmental Medicine, Ernst Moritz Arndt University, Greifswald, Germany; 4 Department of Zoology, Islamia College, Peshawar (CU), Pakistan

**Keywords:** Thyroid Disorders, Epidemiology, Monitoring, Prevalence Trend

## Abstract

**Background:**

Only a few studies like ours have investigated the effect of long-term stable iodine supply on thyroid disorders in a historically iodine-deficient population, but not with a long follow-up time of 10 years.

**Methods:**

Data were derived from two independent population-based cohorts of the Study of Health in Pomerania (SHIP-0 [1997–2001] and SHIP-TREND [2008–2012]) comprising 4308 and 4420 subjects, respectively. Diagnosed thyroid disorders were assessed. Thyroid gland dimensions were examined by ultrasound. Levels of serum thyrotropin (TSH) and autoantibodies to thyroperoxidase (anti-TPO Abs) were measured from blood samples.

**Results:**

Median urinary iodine excretion levels decreased from 123.0 μg/l to 112.0 μg/l (*p* = <0.001) between 2000 and 2010. The prevalence of known thyroid disorders increased from 7.6 % [CI 6.9–8.5] to 18.9 % [CI 17.6–20.1] and of thyroid medication use from 6.2 to 11.1 %. The prevalence of goiter decreased from 35.1 to 29.4 % (*p* = <0.001), while the prevalence of positive anti-TPO Abs decreased from 3.9 to 2.9 % (*p* = 0.022). Median serum TSH levels increased from 0.69 mIU/L to 1.19 mIU/L (*p* = <0.001). Consequently, prevalence of high TSH (mIU/L) increased from 2.6 to 2.9 % (*p* = 0.452), and low TSH (mIU/L) decreased from 6.6 to 6.4 % (*p* = 0.737).

**Conclusion:**

The decreased prevalence of iodine-deficient disorders and a stable prevalence of markers of autoimmune thyroid disorders argue for an improved iodine supply of the adult population in Northeast Germany. In contrast, the prevalence of diagnosed thyroid disorders and the intake of thyroid medication increased, although this might be related to inappropriate therapeutic decisions.

**Electronic supplementary material:**

The online version of this article (doi:10.1186/s12963-016-0111-3) contains supplementary material, which is available to authorized users.

## Background

Until the middle of the 1990s, Germany was considered a region with mild-to-moderate iodine deficiency. The improved iodine fortification program implemented in 1993 elevated the median urinary iodine excretion levels to the lower recommended level [1–3] and reduced goiter prevalence in schoolchildren [2].

The monitoring of iodine fortification programs is important in order to observe the benefits of iodine fortification in populations and to recognize unintended effects early. Ideally iodine deficiency disorder (IDD) prevention should result in a decrease of IDD without significant increase in the prevalence of hypothyroidism and autoimmune thyroid disorders [4, 5]. An increase in the prevalence of hypothyroidism may already be induced by moderate increase in intake of iodine [5, 6]. Thus, iodine fortification of salt should always be introduced cautiously.

The main consequences of long-term iodine deficiency in adults are a high prevalence of goiter, thyroid nodules, and hyperthyroidism. Data from the first cohort of SHIP (SHIP-0), which started a few years after the introduction of the efficient IDD prevention program in Germany, demonstrated a high prevalence of goiter, thyroid nodules, and hyperthyroidism in the general adult population of Northeast Germany [1]. The question arises as to whether a further decade of IDD prevention program is sufficient to observe a decrease in the prevalence of IDD in adults.

Indirectly, the improved iodine supply in Northeast Germany is mirrored by findings from the five-year follow-up examinations of SHIP, in which the normalization rate of baseline goiter was higher than its incident rate [7]. Similar tendencies were observed for thyroid nodules and hyperthyroidism. Also, the incidence rate of positive autoantibodies to thyroperoxidase (anti-TPO Abs) was lower than its normalization rate [7].

With long-term improved iodine supply in Germany we now aim to investigate the change in prevalence of IDD over the last decade, based on two independent cross-sectional studies. Against this background, the rationale of our study was to investigate the change in the prevalence of thyroid disorders between SHIP-0 (1997–2001) and SHIP-TREND (2008–2012). Given a stable iodine supply, we expect a reduction in the prevalence of IDD such as goiter and hyperthyroidism and a nearly stable prevalence of autoimmune thyroid disorders during the past decade. Particularly younger age groups should have benefited from the improved iodine supply.

## Methods

### Study population

The SHIP project consists of two population-based cohorts conducted in West Pomerania, a region in Northeast Germany. The project details are given elsewhere [8, 9]. In SHIP-0, individuals aged 20–79 years were selected from population registries by a two-stage cluster sampling method. The net sample (without migrated or deceased persons) comprised 6265 eligible subjects, of which 4308 (response 68.8 %) participated between 1997 and 2001. A separate stratified random sample of 8826 adults aged 20–79 years was drawn for SHIP-TREND, of which 4420 subjects participated between 2008 and 2012 (response 50.1 %). Random sample selection in age- and sex-strata was facilitated by centralization of local population registries in the Federal State of Mecklenburg/West Pomerania.

The invitation procedure for both cohort studies included three written invitations, phone calls, and one personal contact. Additionally, in SHIP-TREND, temporary examination centres were established in larger communities to facilitate access of older participants to the study in rural areas.

### Assessments

Socio-demographic characteristics and history of diagnosed thyroid disorders were assessed by computer-assisted personal interviews. During data collection, independent auditors regularly reviewed a random sample of 10 % of all interviews for quality. All participants were asked to bring their medications taken seven days prior to the time of examination. Medication data were obtained online using the IDOM program (online drug-database leaded medication assessment) and classified according to the Anatomical-Therapeutic-Chemical (ATC) classification system. Thyroid medication was defined by the ATC code H03.

For SHIP-0, serum TSH levels were analyzed by an immunochemiluminescent procedure (LIA-mat, Byk Sangtec Diagnostica GmbH, Frankfurt, Germany). The functional sensitivity of the assay was 0.03 mIU/L [10]. Coefficients of variation for TSH were 5.0 % at 0.3 mIU/L, 3.7 % at 16.1 mIU/l and 8.9 % over the whole study period. In SHIP-TREND, serum TSH levels were also measured by an immunochemiluminescent procedure (Dimension Vista, Siemens, Eschborn, Germany). The functional sensitivity of the TSH assay was 0.005 mIU/L. The inter-assay coefficients of variations were 2.04 % or 2.20 % for TSH. A method comparison between the two TSH laboratory methods showed only negligible differences [11]. Serum TSH levels were considered low and high based on the reference range established from data for SHIP-0 (0.25 mIU/L–2.12 mIU/L) and SHIP-TREND (0.49 mIU/L–3.29 mIU/L), respectively [10, 11].

Anti-TPO Abs were measured by an enzyme immunoassay in SHIP-0 and SHIP-TREND (VARELISA, Elias Medizintechnik GmbH, Freiburg, Germany). The functional sensitivity of this assay was 1 IU/ml, and the coefficient of variation over the whole study period was 12 %. The anti-TPO Abs status was defined as follows: normal < 60 IU/ml in men and < 100 IU/ml in women; increased ≥ 60 IU/ml in men and ≥ 100 IU/ml in women; positive: > 200 IU/ml in both sexes [1].

Urinary iodine concentrations were measured from spot urine samples by a photometric procedure (Photometer ECOM 6122, Eppendorf, Hamburg, Germany) with Sandell and Kolthoff reaction in SHIP-0 and SHIP-TREND [12]. During the course of the study the inter-assay coefficient of variation for iodine was 4.2 %. Urinary creatinine concentrations were determined with the Jaffé method (SHIP-0: Hitachi 717, Roche Diagnostics, Germany, SHIP-TREND: Dimension Vista, Siemens Healthcare Diagnostics, Eschborn, Germany). The iodine/creatinine ratio was calculated by dividing urinary iodine by urinary creatinine concentrations.

### Thyroid ultrasound

Thyroid ultrasonography was performed in SHIP-0 using an ultrasound VST-Gateway with a 5 MHz linear array transducer (Diasonics, Santa Clara, USA). In SHIP-TREND ultrasonography was performed with a portable device using a 13-MHz linear array transducer (Vivid-I, General Electrics, Frankfurt, Germany). In both studies intra- and inter-observer reliabilities were assessed before the start of the study and semi-annually during the study. For thyroid volume all inter-observer and inter-device variabilities showed mean differences (±2 SD) of < 5 % (<25 %) [13]. Thyroid volume was calculated as length × width × depth × 0.479 (ml) for each lobe [14]. Goiter was defined as a thyroid volume exceeding 18 mL in women and 25 mL in men [15]. The normal thyroid echo pattern was classified as homogeneous. A homogeneous echo pattern with reduced echogenicity was defined as hypoechogenic. Nodular changes exceeding 10 mm in diameter were defined as thyroid nodules.

### Statistical analyses

All analyses were standardized by base-weights to account for different sampling probabilities. In SHIP-TREND, additionally, inverse probability weights for study participation were calculated, which were multiplied with the base-weights. For this, data from a non-responder questionnaire was used, in which 40 % of the non-responders participated. Variables used for the inverse probability weights were marital status, smoking status, general practitioner (GP) visits, general health, diabetes mellitus, and history of myocardial infarction and stroke.

Continuous variables were described by the median and its 95 % confidence interval (CI). Categorical variables were described by the prevalence and its 95 % CI. Differences in median levels between SHIP-0 and SHIP-TREND were tested by median regression models; prevalence differences between SHIP-0 and SHIP-TREND were tested by Poisson regression models. A *p* < 0.05 was considered as statistically significant. All analyses were carried out with Stata 13.1 (Stata Corporation, College Station, TX, USA).

## Results

The median age of the participants was 50 years in SHIP-0 and 53 years in SHIP-TREND. The frequency of males was 49.1 % in SHIP-0 and 48.5 % in SHIP-TREND.

There were 36 individuals excluded from analysis in SHIP-0 because of missing data regarding diagnosed thyroid disorders. Similarly, in SHIP-TREND there were 13 individuals with missing information on known thyroid disorders.

### Diagnosed thyroid disorders and medication

**Fig. 1:**
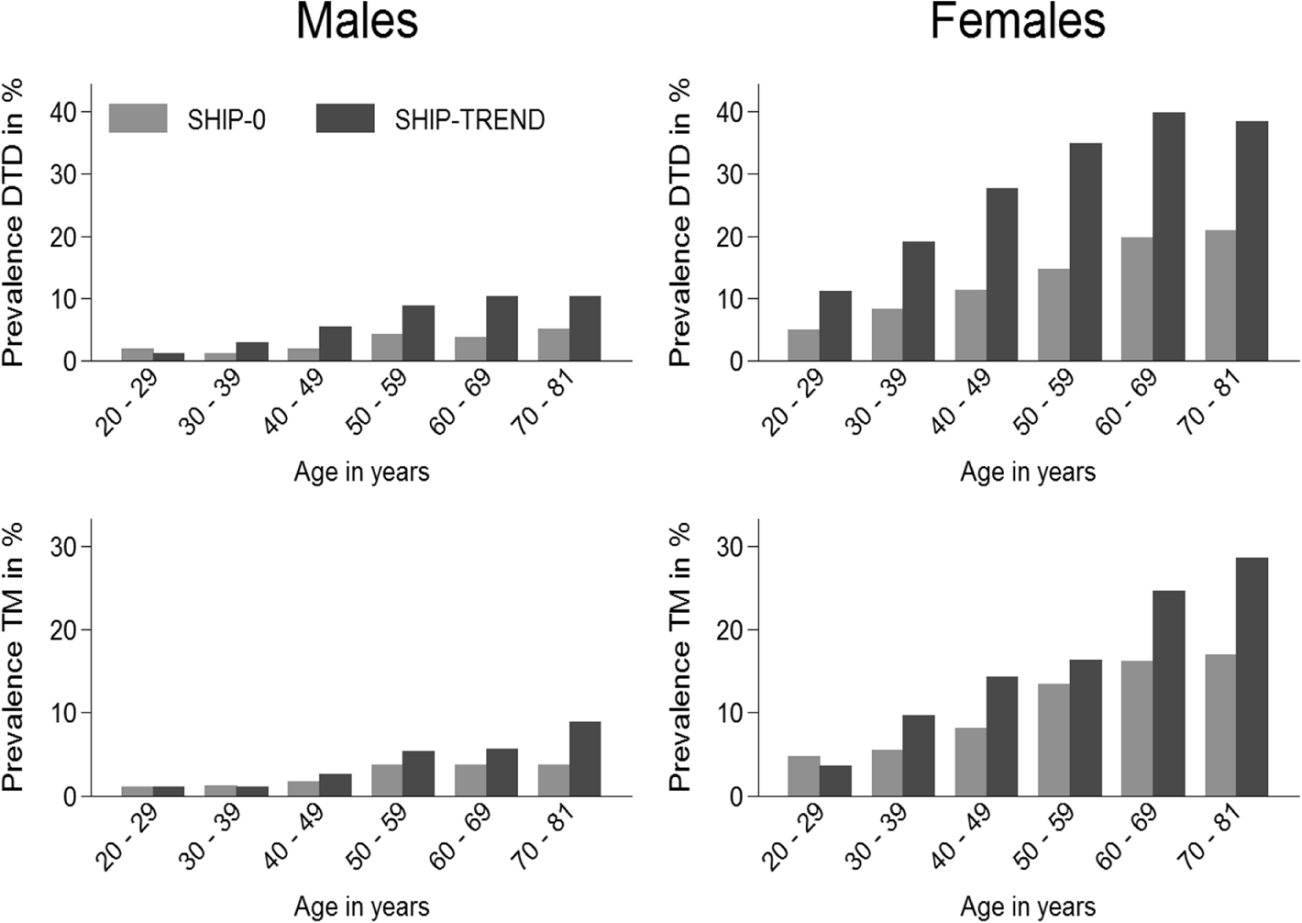
Age- and sex-stratified change in prevalence of diagnosed thyroid disorders (DTD) and thyroid medication (TM) between SHIP-0 and SHIP-TREND

There was an increase in the prevalence of diagnosed thyroid disorders between SHIP-0 and SHIP-TREND from 7.6 % [CI 6.9–8.5] to 18.9 % [CI 17.6–20.1]. This increase was similar for both genders (in males from 12.6 % [CI 11.2–14.2] to 30.5 % [CI 28.4–32.6]; in females from 2.4 % [CI 1.9–3.2] to 6.5 % [CI 5.5–7.7]). The increase in prevalence of diagnosed thyroid disorders was observed in all age- and sex-specific strata (Fig. 1).

The prevalence of thyroid medication intake increased from 6.2 % [CI 5.5–7.0] in SHIP-0 to 11.1 % [CI 10.1–12.2] in SHIP-TREND. This increase was more pronounced in males (from 2.1 % [CI 1.6–2.8] to 4.1 % [CI 3.3–5.1]) than in females (from 10.2 % [CI 18.9–11.7] to 17.8 % [CI 16.0–19.7]). In both studies thyroid hormone therapy was the most frequent thyroid medication used. We observed an increase in thyroid hormone therapy from 4.4 % [CI 3.8–5.1] to 10.4 % [CI 9.4–11.4]. This increase was similar in males (from 1.5 % [CI 1.1–2.1]; to 3.8 % [CI 3.1–4.8]) and females (from 7.1 % [CI 6.1–8.4]; to 16.6 % [CI 14.9–18.4 %] and stronger in the elderly (Fig. 1).

For further analyses of thyroid-related measurements we excluded all individuals with diagnosed thyroid disorders and thyroid medication, resulting in study populations of 3850 individuals (2017 women) in SHIP-0 and 3564 individuals (1975 women) in SHIP-TREND.

### Urinary iodine and iodine-to-creatinine excretion levels

**Fig. 2:**
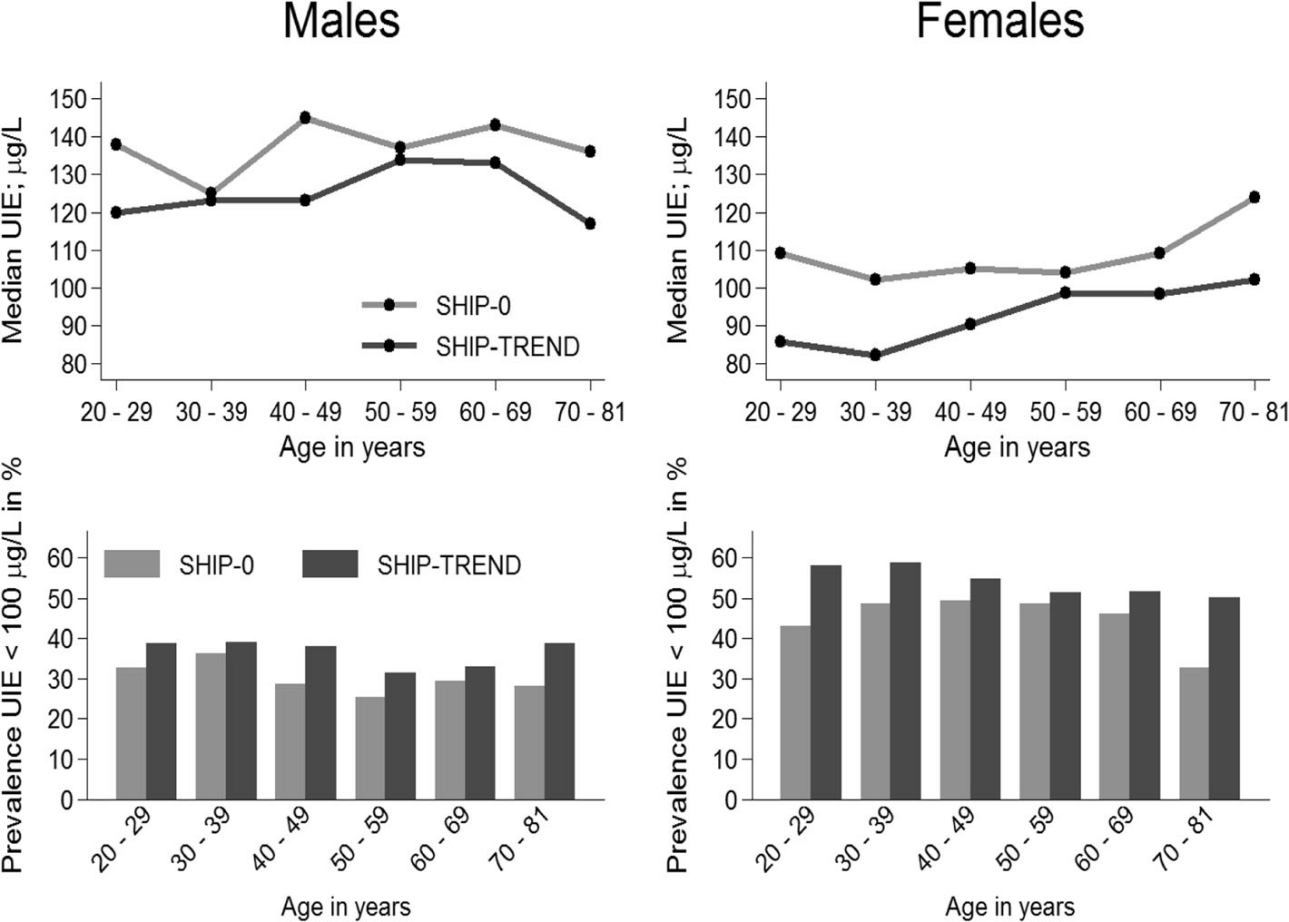
Age- and sex-stratified change in urinary iodine excretion (UIE) levels between SHIP-0 and SHIP-TREND

There was a significant decrease in median urinary iodine excretion levels between SHIP-0 and SHIP-TREND, which was more pronounced in females than in males (Table 1). In SHIP-TREND median urinary iodine excretion levels remained almost stable among all age groups in males, but increased with advancing age in females (Fig. 2). Prevalence of median urinary iodine excretion levels <100 μg/L increased from SHIP-0 to SHIP-TREND (Table 1). This difference was stronger in females compared to males and particularly present among youngest and oldest females (Fig. 2). The median iodine-to-creatinine ratio declined in all sex- and age-groups between SHIP-0 and SHIP-TREND with stronger decrease in females than in males (Table 1).

**Table 1:** Change in thyroid characteristics between SHIP-0 (1997–2001) and SHIP-TREND (2008–2012)

	All			Males			Females		
	SHIP-0	SHIP-TREND	p*	SHIP-0	SHIP-TREND	p*	SHIP-0	SHIP-TREND	p*
Iodine, μg/l	123.0 (122.5; 123.5)	112.0 (111.5; 112.5)	<0.001	134.0 (133.2; 134.8)	126.0 (125.5; 126.5)	<0.001	108.0 (107.2; 108.8)	91.9 (91.4; 92.4)	<0.001
Iodine to creatinine ratio, μg/g	130.1 (129.8; 130.4)	113.7 (113.3; 114.0)	<0.001	116.2 (115.7; 116.8)	105.8 (105.4; 106.2)	<0.001	152.2 (151.4; 153.0)	128.0 (127.2; 128.8)	<0.001
Iodine < 100 μg/L, %	37.7 (36.0; 39.5)	43.9 (42.0; 45.7)	<0.001	30.5 (28.2; 32.9)	36.2 (33.9; 38.6)	0.001	45.6 (43.0; 48.3)	53.8 (51.0; 56.6)	<0.001
Thyroid volume, ml	18.41 (18.36; 18.46)	18.27 (18.24; 18.30)	<0.001	21.79 (21.71; 21.87)	20.39 (20.35; 20.44)	<0.001	15.31 (15.26; 15.36)	15.43 (15.40; 15.47)	<0.001
Goiter, %	35.1 (33.5; 36.7)	29.4 (27.8; 31.0)	<0.001	36.5 (34.2; 38.8)	26.6 (24.7; 28.7)	<0.001	33.7 (31.5; 36.0)	32.9 (30.4; 35.6)	0.676
Thyroid nodules, %	18.5 (17.3; 19.8)	30.5 (29.0; 32.1)	<0.001	14.8 (13.2; 16.7)	24.4 (22.5; 26.4)	<0.001	22.5 (20.7; 24.3)	38.4 (35.9; 41.0)	<0.001
Hypoechogenic thyroid pattern, %	5.4 (4.8; 6.2)	3.7 (3.1; 4.4)	0.001	1.8 (1.3; 2.4)	2.6 (1.9; 3.4)	0.113	9.5 (8.1; 11.0)	5.2 (4.1; 6.5)	<0.001
Increased anti-TPO antibodies, %	6.7 (5.8; 7.6)	5.2 (4.5; 6.0)	0.013	3.7 (2.9; 4.8)	3.7 (2.9; 4.7)	0.994	9.9 (8.5; 11.5)	7.1 (5.9; 8.6)	0.007
Positive anti-TPO antibodies, %	3.9 (3.2; 4.6)	2.9 (2.4; 3.5)	0.022	1.3 (0.9; 1.9)	1.4 (1.0; 2.1)	0.677	6.7 (5.6; 8.1)	4.7 (3.7; 6.0)	0.017
TSH, mIU/L	0.69 (0.68; 0.70)	1.19 (1.18; 1.20)	<0.001	0.68 (0.67; 0.69)	1.16 (1.15; 1.17)	<0.001	0.71 (0.70; 0.72)	1.24 (1.23; 1.25)	<0.001
Increased TSH, %	2.6 (2.1; 3.2)	2.9 (2.4; 3.6)	0.452	1.6 (1.1; 2.4)	2.4 (1.7; 3.3)	0.122	3.7 (2.9; 4.8)	3.6 (2.7; 4.8)	0.960
Decreased TSH, %	6.6 (5.8; 7.5)	6.4 (5.6; 7.3)	0.737	6.7 (5.6; 8.1)	6.4 (5.4; 7.6)	0.684	6.5 (5.4; 7.7)	6.4 (5.2; 7.8)	0.940

Dichotomous data are presented as percentage and 95 % confidence interval; continuous data as median and 95 % confidence; all analyses are weighted by sampling weights; *p-values for differences between the studies are taken from Poisson (dichotomous data) and median regression models (continuous data)

### Thyroid volume and nodules

The median thyroid volume remained similar between SHIP-0 and SHIP-TREND in the whole study population. In males, however, there was a considerable decrease in the thyroid volume between SHIP-0 and SHIP-TREND, while in females; the thyroid volume did not substantially differ between the two studies (Table 1).

**Fig. 3:**
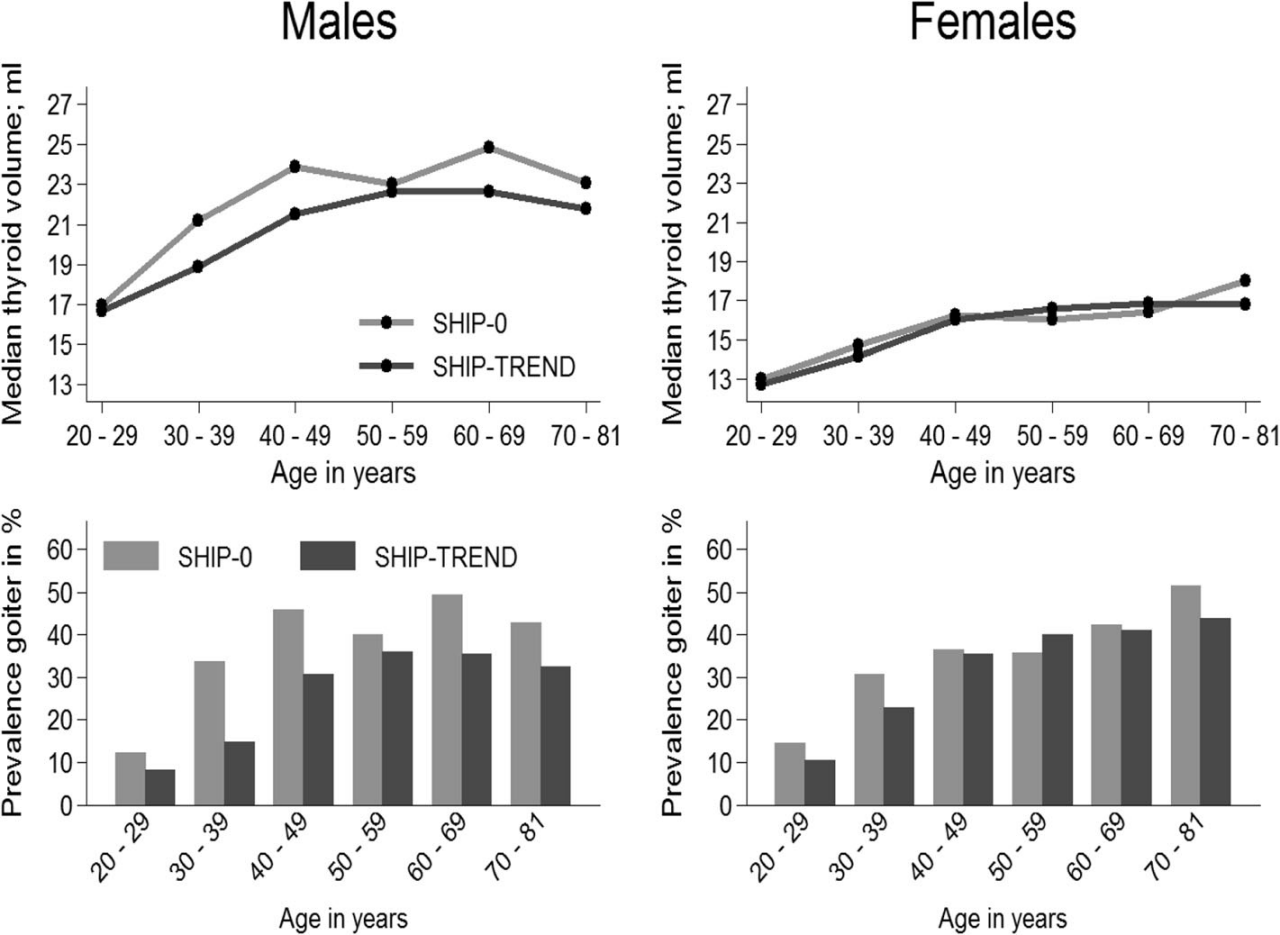
Age- and sex-stratified change in thyroid volume and goiter prevalence between SHIP-0 and SHIP-TREND

Goiter prevalence decreased significantly between SHIP-0 and SHIP-TREND. Again, this decrease was more pronounced in males than in females and was observed in nearly all age and sex groups except for 50–59 years old females (Fig. 3).

The prevalence of thyroid nodules increased between SHIP-0 and SHIP-TREND (Table 1). This increase was more pronounced in females than in males. There was an age-dependent increase in prevalence of thyroid nodules with highest values in 60–69 year old males and 50–59 year old females.

### Hypoechogenic thyroid pattern and anti-TPO Abs

**Fig. 4:**
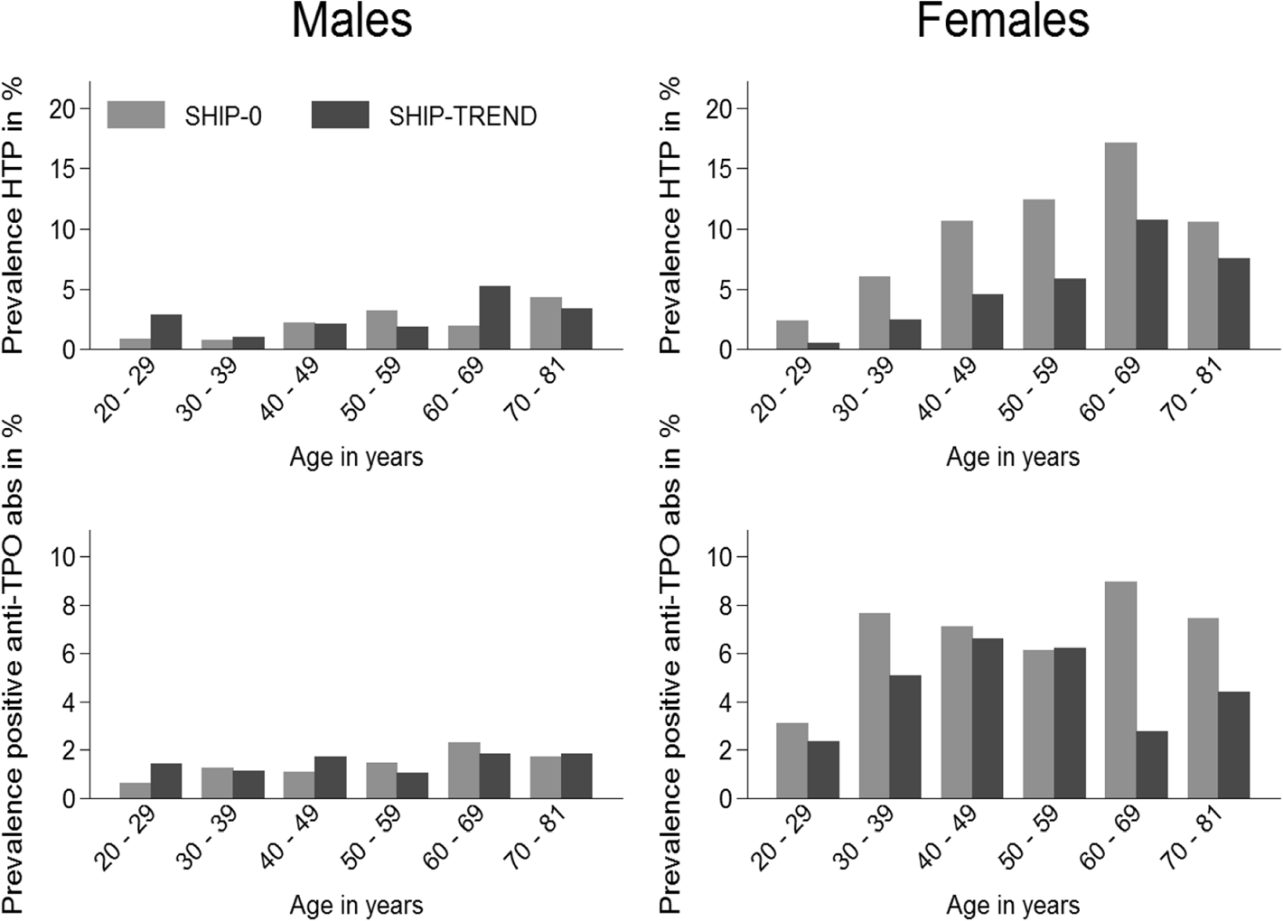
Age- and sex-stratified change in prevalence of hypoechogenic thyroid pattern (HTP) and positive anti-TPO antibodies (anti-TPO Abs) between SHIP-0 and SHIP-TREND

The prevalence of hypoechogenic thyroid pattern decreased from SHIP-0 to SHIP-TREND. This decrease was present predominantly in females, while in males the prevalence remained almost stable. Within the gender and age groups the strongest decrease was observed in the age decade of 60–69 years in males and 40–69 years in females (Fig. 4).

Similarly, the prevalence of increased anti-TPO Abs and positive anti-TPO Abs decreased from SHIP-0 to SHIP-TREND in the whole study population (Table 1). While in males the prevalence of increased anti-TPO Abs and positive anti-TPO Abs was stable between the studies, there was a decrease in the prevalence from SHIP-0 to SHIP-TREND in females. No clear trend of positive anti-TPO Abs prevalence was observed over age groups in both studies and sexes (Fig. 4).

### Thyroid function

Median serum TSH levels increased significantly from SHIP-0 to SHIP-TREND, resulting in a right shift of the serum TSH level distribution (Additional file 1: Figure S1). This increase was stronger in females than in males (Table 1).

**Fig. 5:**
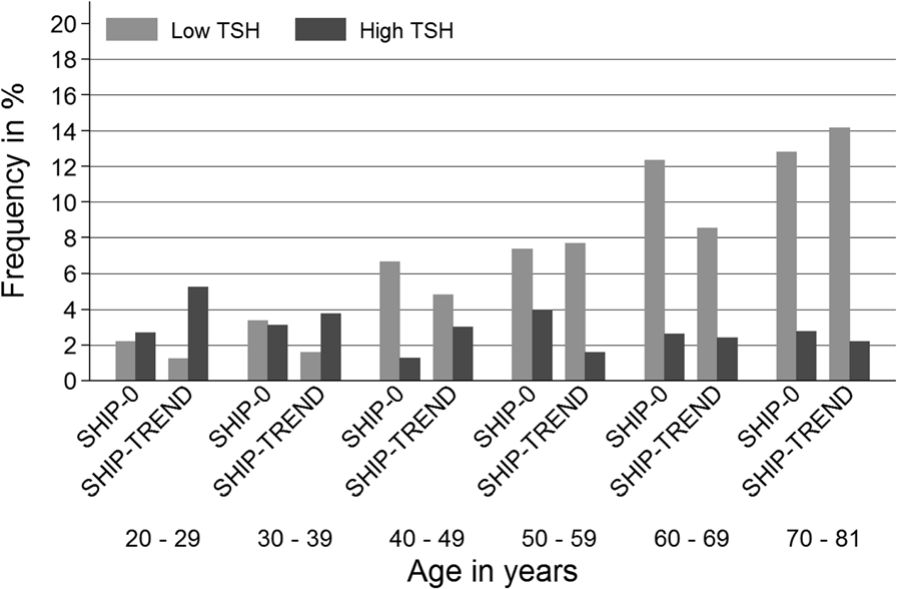
Age-stratified frequency distribution of low and high TSH between SHIP-0 and SHIP-TREND

The prevalence of high serum TSH levels remained almost stable between SHIP-0 and SHIP-TREND. This increase was seen in males only (Table 1). In SHIP-0 there was no age-dependent tendency in prevalence of increased serum TSH levels, but in SHIP-TREND mainly younger individuals between 20 and 49 years were affected (Fig. 5). Likewise, the prevalence of low TSH remained nearly stable between SHIP-0 and SHIP-TREND, which was also seen in both sexes (Table 1) (Fig. 5).

## Discussion

In the present study we investigated the effects of long-term IDD prevention by comparing the prevalence of thyroid disorders between 2000 and 2010 (7 and 17 years after the initiation of the iodine fortification program, respectively) based on population-based data from the same study region of Northeast Germany.

We demonstrate a decrease in the prevalence of goiter in men and an increase in median serum TSH levels. In contrast, the prevalence of thyroid nodules increased, but this could most likely be explained by the better resolution of the thyroid ultrasound device in SHIP-TREND than in SHIP-0 [16]. Our data, therefore, argue for a decline of IDD during the last decade, while the prevalence of thyroid diseases related to iodine repletion such as hypothyroidism or positive anti-TPO Abs was stable or even decreased during that time period.

In contrast to the generally expected trend of subclinical thyroid disorders and, of very important note, self-reported diagnosed thyroid disorders and treatments with thyroid medication increased. This is clearly against the aims of an IDD prevention program, but the question is whether this finding can be related to the improved iodine status of the Northeast German population. We assume that a few alternative factors may explain this phenomenon.

First, an inadequate prescription of thyroxine due to therapeutic misjudgment may not be ruled out. Data from the Framingham study demonstrated a high prescription rate of thyroxine in elderly individuals for non-thyroid indications [17]. In Europe there are also examples for misuse of thyroxine. Findings of the DanThyr study from Denmark showed that levothyroxine might have been prescribed to the wrongly diagnosed thyroid diseases such as hypothyroidism and hyperthyroidism and their subclinical forms [18]. Second, the diagnosis of thyroid disorders depends on the definition of TSH reference intervals. For example, diagnosis of subclinical hypothyroidism critically depends on the upper TSH reference limit [19]. As demonstrated in our study there is a substantial increase in median serum TSH levels between SHIP-0 and SHIP-TREND. In consequence, the formerly low upper reference limit established with data from SHIP-0 [10], still applied in clinical practice, does not adequately represent the current situation and might have led to an increased rate of thyroxine prescription. Therefore, we recently established a new reference interval based on data from SHIP-TREND, which clearly indicates that the upper reference limit has to be raised substantially in our study region [11]. Third, inappropriate treatments may be responsible for the increase in prevalence of diagnosed thyroid disorders and thyroid medication use. Therapeutic decisions may commonly be based on TSH reference values only, which according to recent guidelines is considered a common error [20], as it neglects the symptoms and complaints of the patient [21]. Finally, increased awareness about thyroid disorders in recent years, leading to the frequent use of thyroid laboratory tests, may have contributed to the higher prevalence of diagnosed thyroid disorders and consequently, use of thyroid medications. Additionally, the increased prescription of thyroxine for the thyroid nodules in SHIP-TREND may not be considerable, and further the data are not comparable for the different devices used in the two cohorts. However, owing to the increased prevalence of thyroid nodules in SHIP-TREND, this assumption may not be ruled out entirely.

Iodine intake in a population is the major determinant of thyroid disorders. Even a small change in the level of iodine intake in a population can change the frequency of thyroid-related disorders [22]. The median iodine excretion levels throughout Germany in recent years are reportedly declining [23]. The German health interview and examination survey for adults (DEGS 2008–2011) revealed a drastic decrease (61 μg/L) in iodine excretion levels, comparing the results (117 μg/L) to the previously conducted German health interview and examination survey for children and adolescents (KiGGS 2003–2006) [3]. Though in comparison to DEGS, in our study the median iodine excretion levels decreased slightly between SHIP-0 and SHIP-TREND, but importantly without detrimental effects. Median iodine excretion levels were lower in females (particularly in younger women) than in males in both studies. This difference may be explained by the higher intake of fluids by women compared to men [24]. A greater than normal intake of water will increase the urine volume, leading to lower urinary creatinine and lower iodine concentrations, but usually will not reduce the amount of creatinine excreted daily [25]. Thus, the iodine/creatinine ratio adjusts for differences in fluid intake and may provide a better estimate of the true iodine intake in young females [24, 26]. This argument was supported by the DEGS conclusions [23]. On the other hand, there was a marginal but significant increase in median thyroid volume in women, while goiter prevalence remained nearly unchanged. In contrast, thyroid volume and goiter prevalence decreased in men. This sex-specific difference may be explained by a suboptimal iodine status in women compared to men.

When improving iodine supply there is always a risk to overcompensate, which might result in a high prevalence of autoimmune thyroid disorders. A sudden and strong increase in iodine intake may provoke the formation of thyroid antibodies [4, 5, 27]. In our study, there is no indication of a substantial increase in autoimmune thyroid disorders parallel to the iodine fortification program, which is in line with the longitudinal findings of the SHIP cohort [7]. We observed a decrease in the prevalence of elevated and positive anti-TPO Abs between SHIP-0 and SHIP-TREND. Previous trend studies have focused on the influence of sudden or periodic increase in iodine supply among the population and of thyroid autoimmunity [28, 29]. In contrast, we studied the effect of consistently optimal iodine supply long-term on the same outcome. In a Chinese study conducted four years after initiation of iodine fortification, [5] no difference in the five-year incidence rates of thyroid autoimmunity was detected. In contrast, a Danish registry study, studying all new cases of thyroid autoimmunity, four to five years after mandatory iodine fortification, demonstrated an increase in incidence (32.0 %) compared to baseline (20.0 %). However, this increase was in response to increase of iodine intake from previous moderate and mild to mandatory iodine intake in an overall cohort [28]. Similarly, a marked increase in incidence of elevated anti-TPO Abs was observed in a study from Slovenia 10 years after the increase in iodine fortification, leading to an improvement of the iodine supply from mildly deficient to sufficient [29].

In our study, prevalence of increased and positive anti-TPO Abs was higher in females than in males, which is in concordance with other studies [5, 30]. In the Danish study, [28] prevalence of positive anti-TPO Abs showed an age-dependent increase with time, which was not observed in our study, as it was highest in middle-aged (30–60 years) women. However, the age-dependent prevalence of autoimmune thyroid disorders is strongly related to the iodine repletion history or sudden increase in iodine intake in a region [31]. Hence, the decrease in prevalence of elevated and positive anti-TPO Abs argues for an optimal but slightly declining iodine supply in our study region over the past two decades.

It is evident that the current and historical iodine supply to a region determines the distribution of TSH values in the population [1]. If the region is iodine-deficient the TSH reference interval tends to be lower than in regions with higher iodine supply [10, 32–35]. However, when a region is moving toward sufficiency from deficiency, initially the TSH distribution may shift toward the left, as was demonstrated in the SHIP-0 population [10]. With the persistence of improved iodine supply for several years, the TSH value distribution for the general population shifts toward the right [11]. Even the TSH reference range established during the transition phase from deficiency to sufficiency may not reflect the current situation [11]. In our study this shift cannot be explained by different populations, sex, and current smoking, as these distribution were quite similar between SHIP-0 and SHIP-TREND [11]. The association between serum TSH levels and BMI has been previously reported [36, 37] and although in SHIP-TREND the median BMI was slightly higher than in SHIP-0, the detected differences in BMI might be too small to explain this magnitudinal change in TSH values [11]. We assume that the change in TSH is mainly related to the improved iodine supply. A similar association has been previously reported for the Danish population [4]. In contrast to the studies from the US, we could not observe an increase in TSH levels with age [38, 39]. It may be explained by the history of iodine deficiency in our region and more than 60 years of sufficiency in the US population [40].

Iodine repletion in populations may result in a higher prevalence of hypothyroidism [41]. A Chinese study, also conducted in a historically iodine-deficient region after iodine fortification, showed, in agreement with our study, no increase in incidence of overt hypothyroidism. However, there was a slight increase in subclinical hypothyroidism in that study [5]. In contrast, based on hospital registry data, a Danish study demonstrated an increase in incidence of hypothyroidism from 38/100,000^.^yr to 48.7/100,000^.^yr after the increase of iodine intake from moderate to mild [6]. Differences in results between our study and the Danish study can be mainly explained by two factors. First, in the Chinese study and in SHIP, the iodine level was almost consistent throughout the study period [5, 42], while in the Danish study a periodic increase in iodine intake was introduced [6]. Second, in our study and in the Chinese study the TSH reference range was adapted to the changing iodine status of the study population, which was not done in the Danish study [5, 43]. Furthermore, the Chinese study concluded that a shorter follow-up time may be responsible for non-recording of overt hypothyroidism because of its long latency period. In contrast to the Danish and the Chinese studies, which had follow-up periods of eight and five years, our follow-up was conducted 10 years after baseline [5]. Thus, the different follow-up periods are no explanation for the different findings across these studies. In our study there is equilibrium in prevalence of hypo- and hyperthyroidism defined by increased and decreased serum TSH levels in SHIP-TREND using regional reference limits, which might reflect adequate iodine supply over the last 19 years in our study region.

Strengths of our study are the population-based design of both studies, which were conducted in the same study region one decade apart. In both SHIP studies, examinations were executed in the same laboratory with a common approach to high standardization of measurements. A limitation is that in SHIP-0 and SHIP-TREND, different thyroid ultrasound devices were used, which particularly led to a higher detection rate of thyroid nodules in SHIP-TREND. Another limitation of our study is that we have not measured thyroglobulin (Tg) antibodies in the SHIP studies, which hampers the interpretation of our findings, since an increase in iodine supply may lead to an increase in thyroglobulin (Tg) antibody concentrations [44]. However, the slight reduction in prevalence of positive anti-TPO Abs may indicate that the prevalence of autoimmune thyroid disorders is stable in our study region. A further limitation is that we have no data on fT3 and fT4 in SHIP-TREND, making it impossible to distinct overt and subclinical forms of thyroid dysfunction. Additionally, results may be affected by selection bias as the participation rate decreased to 50.1 % in SHIP-TREND as compared to 68.8 % in the baseline cohort, though inverse probability weights were calculated for SHIP-TREND study participation and used multiplicatively to the base-weights to reduce selection bias.

## Conclusion

In conclusion, the improved iodine supply over the past two decades in Germany is paralleled by a reduction in prevalence of IDDs. Furthermore, there was no increase in prevalence of markers of autoimmune thyroid disorders, arguing for an optimal iodine supply among the general adult population in Northeast Germany; however, the slight decrease in urinary iodine concentrations may be alarming. In contrast to this, the prevalence of diagnosed thyroid disorders and the intake of thyroid medication increased in that time period, which might be related to inappropriate therapeutic decisions and more frequent use of thyroid laboratory test in recent years. Thus, we recommend that therapeutic decisions should be made with caution, based on regional TSH reference ranges, the test’s prognostic value, and compliance with treatment.

## Additional file

Additional file 1: Figure S1. Change in the distribution of serum TSH values between SHIP-0 and SHIP-TREND. (DOCX 340 kb)

## Abbreviations

anti-TPO: bs Autoantibodies to thyroperoxidase
ATC: Anatomical-Therapeutic-Chemical
CI: Confidence interval
GP: General practitioner
IDD: Iodine deficiency disorder
IDOM: Online drug-database leaded medication assessment
SHIP: Study of Health in Pomerania
