# Supplementary figures and images for: Monitoring the prevalence of thyroid disorders in the adult population of Northeast Germany
Source: Popul Health Metr. 2016 Nov 8;14:39. doi: 10.1186/s12963-016-0111-3 (PMC5101821; doi:10.1186/s12963-016-0111-3)

**Additional file 1: Figure S1.**


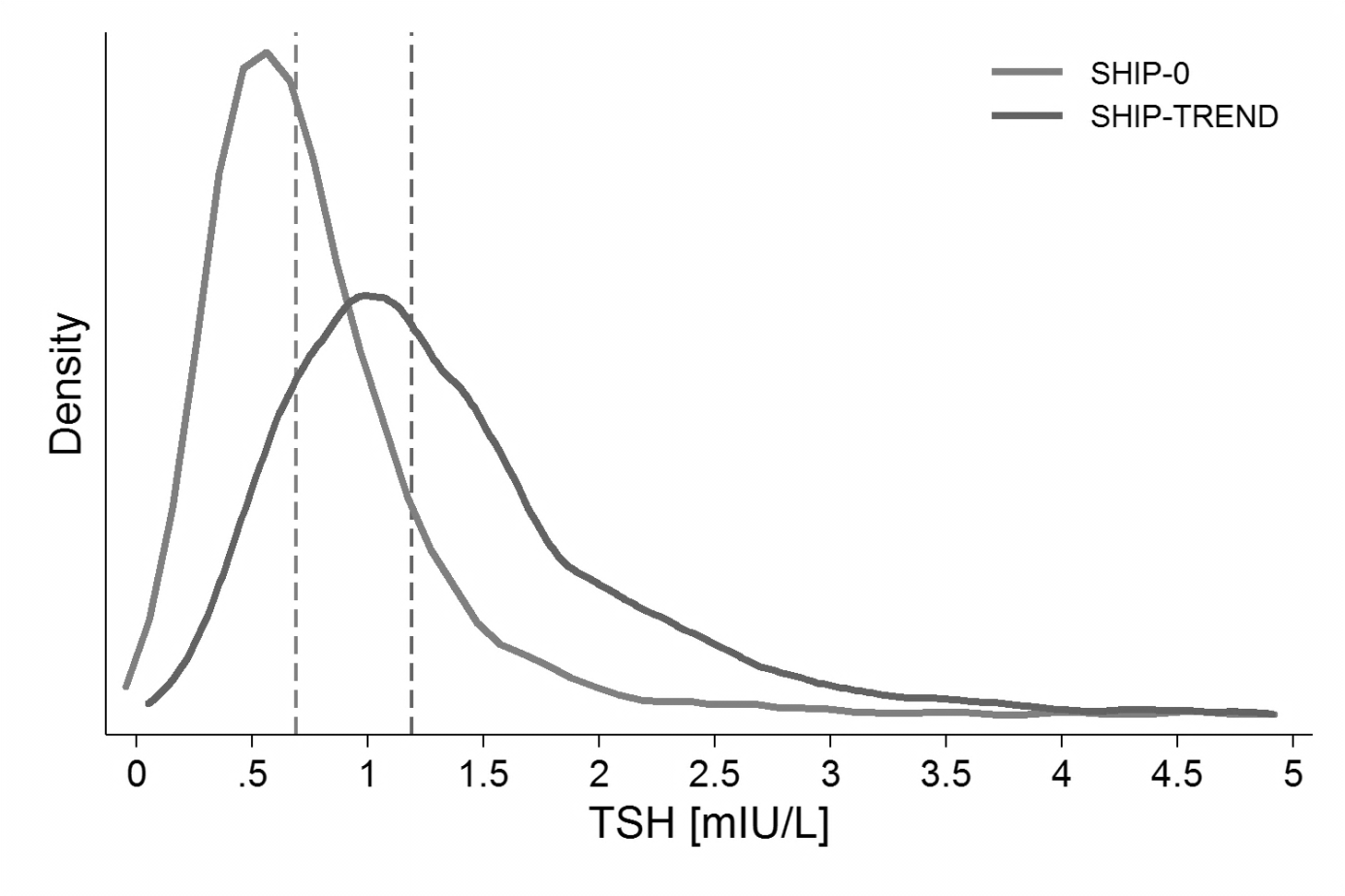

Supplement: Additional file 1: Figure S1. — Change in the distribution of serum TSH values between SHIP-0 and SHIP-TREND. (DOCX 340 kb) [file 12963_2016_111_MOESM1_ESM.docx]
